# A new measure of predictive ability for survival models

**DOI:** 10.1186/1745-6215-12-S1-A138

**Published:** 2011-12-13

**Authors:** Babak Choodari-Oskooei, Patrick Royston, Mahesh KB Parmar

**Affiliations:** 1 MRC Clinical Trials Unit, London, WC2B 6NH, UK

## 

In clinical research, an understanding of prognostic factors is important in the design and analysis of clinical trials and retrospective reviews of clinical experience. The results of prognostic factor studies are usually summarized in the form of statistics resulting from statistical significance testing, i.e. estimated parameters, confidence intervals, and p-values. These statistics do not inform us whether prognostic factor information will lead to substantial improvement in the prognostic assessment. Predictive ability measures can be used for this purpose since they provide important information about the practical significance of prognostic factors. *R*^2^-type indexes are the most familiar forms of such measures in survival models, but they all have limitations and none is widely used.

Bura and Gastwirth (2001) [1] proposed a new predictive ability measure, named total gain (TG), for a logistic regression model. TG is based on the binary regression quantile plot, otherwise known as the predictiveness curve, which was first proposed by Copas (1999) [2]. Gu and Pepe (2009) [3] showed that TG is related to the ROC summary index, but it does not have the reported shortcomings of the ROC index.

In this paper, we extend the proposed TG measure to survival models and explore its properties using simulations and real data. In survival models, the TG statistic is a non-negative, unitless measure of the total cumulative distance between the average survival probability, as expressed by the Kaplan-Meier (KM) estimates of the survival probability, at a fixed time point and the estimated survival probabilities from a given model. Standardised TG ranges from 0 (no explanatory power) to 1 (‘perfect’ explanatory power).

In our simulation studies, we investigated the impact of censoring, covariate distribution and influential observations on the measure. The results of our simulations show that unlike most of the other *R*^2^-type predictive ability measures, TG is independent of censoring and follow-up time. TG also increases as the effect of a covariate increases, but it is adversely affected by the categorisation of continuous prognostic factors. Finally, we applied TG to quantify the predictive ability of prognostic models developed in several disease areas. On balance, although TG lacks the intuitive interpretation of the explained variation measures, our results indicate that the estimates of the measure are within the reasonable range of the estimates of explained variation measures and can be recommended as an alternative measure to quantify the predictive ability in survival models.
